# Mr. Shaul, on Spasmodic Affection

**Published:** 1800-07

**Authors:** Edmund Shaul

**Affiliations:** Docking, Norfolk


### *To the Editors of the Medical and Phyſical Journal.*

GENTLEMEN,

IF the following ſingular caſe, from its ſucceſsful termination, (which appears to have been effected principally by the exhibition of the Digitalis, and may, probably, merit the attention of practitioners in ſo dreadful a malady) ſhould be deemed worthy a place in your periodical publication, I offer it to your ſervice; and am,

GENTLEMEN,

With very great reſpect,

Your moſt humble ſervant,

EDMUND SHAUL.

*Docking, Norfolk,*
*June* 6, 1800.

---

S. O. a young women aged twenty-one years, of low ſtature, and of a feeble, florid habit, having over-exerted herſelf in walking or running ten miles in a ſhort ſpace of time; in conſequence of which, ſhe fainted, and became extremely languid and debilitated, being three months advanced in pregnancy. Symptoms of irritation, with great tendency to abortion, immediately ſupervened; but, upon examination, there was no appearance of dilatation of the os tincæ; and the vagina gina was very much contracted, ſcarcely admitting the introduction of the fingers. She was ordered to keep herſelf in a horizontal poſition, and to abſtain from all heating liquors; an anodyne mixture was alſo adminiſtered, compoſed of the mixtura camphorata. tinct. caſtor & tinct. opii; a doſe of which was ordered to be taken every three hours. But the pains recurring at times, accompanied with a general hyſteria, I was ſent for at twelve o'clock at night, and found her labouring under an increaſe of ſymptoms, but ſtill there was no dilatation of the os tincæ. I then gave her a draught with tinct. opii. gts. 40, and æther. vitriol. increaſing the tinct opii. gradually. This affording a temporary relief, ſhe took ſome nouriſhment, and was diſpoſed to ſleep; but in an hour afterwards ſhe became violently convulſed, and every ſymptom of the moſt dreadful ſpaſmodic affection, ever witneſſed by any medical practitioner, now came on. The opiſthotonos and emproſthotonos alternately ſucceeding each other, the head and extremities were bent backwards ſo as almoſt to meet: ſoon after an action the reverſe of this took place; her chin and knees were contracted together, the muſcles of the face and her eyes were violently diſtorted, the latter being almoſt protruded from their ſockets; and during the paroxyſms ſhe frequently attempted to bite herſelf. In ſhort, every muſcle of her ſyſtem appeared more or leſs to labour under ſpaſmodic action.

In this deſperate ſtate I repeated the tinct. opii with ſome æther; but no relief being obtained, I ordered it to be given to her every two hours, and an emollient clyſter was alſo injected. This mode of treatment not anſwering my expectations, I ſent for a medical friend to ſee this ſingular and deplorable caſe; and as our preſent plan ſeemed to afford ſo unſuccefsful a proſpect, another expedient was adopted: Twelve ounces of blood were taken from her arm, and I ordered the abdomen and extremities of the body to be rubbed with an embrocation, conſiſting of the linimentum ſaponis and tinct. opii aa. ℥j; and 100 drops of the tinct. opii to be given internally, with æther. vitriol. ʒiſs. Twenty grains of the pil. gummoſæ were alſo adminiſtered every three hours, and I recommended her to go into a warm bath for ten minutes. The ſpaſms ſtill continuing, but with a leſſer degree of violence, Dr. Redfearn, a phyſician at Lynn, was ſent for to viſit her. After ſome deliberation, and having communicated to him the different remedies I had made uſe of, he ordered me to give her inſtantly 200 drops of the tinct. opii, and the following medicines were preſcribed:

℞. Emulſionis camphorat. ℥iv; tinct. opii, tinct. caſtorei aa ℥ſs. Miſce. fiat enema ſtatim injiciendum.

 ℞. Tinct.

℞. Tinct. opii, ætheris vitriol. aa ℥j; ol. fuccini ʒij. Fiat embrocatio toto corpori applicand. poft balneum tepidum.

The following draughts were alfo prefcribed:

℞. Emulfionis camphorat. ℥ifs; tinct. digitalis purp. gt. xl; tinct. opii gt. 160; ætheris vitriol. ʒj. M. Fiat hauftus tertiâ quaque horâ fumendus.

The tinctura digitalis was increafed gradually to 70 drops at one dofe. At laft more favourable fymptoms appeared; the fpafms were mitigated, and fhe paffed a tolerably eafy night without being affected by them. However, on the following morning, a return of the former fymptoms feemed to be rapidly coming on; her eyes were very much diftorted, and the Doctor ordered the enema to be repeated with ʒij of the gum affafœtida added to it; and a bolus, compofed of the different articles fubjoined, to be inftantly given.

℞. Opii purif. gt. vij; zinci calcinat. gt. ifs; pul. digit. purp. gt. ij; piperis cayenfis gt. iij. Syrup. s. s. Fiat bolus ftatim fumendus.

Soon after the above medicine was given, a general relaxation of the whole fyftem fucceeded, attended with naufea, and a total ceffation of fpafmodic action. Being extremely debilitated, a diet of wine, eggs, and coffee was prefcribed, with as much nutriment in various forms as could be got down. The tinct. opii was now diminifhed gradually, and vegetable and mineral tonics were adminiftered. By continuing this regimen her health became perfectly eftablifhed, and fhe has ever fince remained entirely free from any return of fpafmodic action.

The quantity of the tinct. opii, taken internally at different times, in the courfe of three days, was two thoufand two hundred and ten drops; one ounce was injected per anum, and two ounces and a half applied externally.

